# Fragment-Based Whole Cell Screen Delivers Hits against *M. tuberculosis* and Non-tuberculous Mycobacteria

**DOI:** 10.3389/fmicb.2016.01392

**Published:** 2016-09-07

**Authors:** Wilfried Moreira, Jia Jie Lim, Si Ying Yeo, Pondy M. Ramanujulu, Brian W. Dymock, Thomas Dick

**Affiliations:** ^1^Department of Microbiology and Immunology, Yong Loo Lin School of Medicine, National University of SingaporeSingapore, Singapore; ^2^Department of Pharmacy, National University of SingaporeSingapore, Singapore; ^3^Centre for Life Sciences, Life Sciences Institute, National University of SingaporeSingapore, Singapore; ^4^BSL3 Core Facility, Centre for Translational Medicine, Yong Loo Lin School of Medicine, National University of SingaporeSingapore, Singapore

**Keywords:** fragments, poly-pharmacology, tuberculosis, NTM, *M. abscessus*, *M. avium*

## Abstract

Reactive multi-target ‘fragment drugs’ represent critical components of current tuberculosis regimens. These compounds, such as pyrazinamide, are old synthetic antimycobacterials that are activated inside *Mycobacterium tuberculosis* bacilli and are smaller than the usual drug-like, single-target molecules. Based on the success of small ‘dirty’ drugs in the chemotherapy of tuberculosis, we suggested previously that fragment-based whole cell screens should be introduced in our current antimycobacterial drug discovery efforts. Here, we carried out such a screen and characterized bactericidal activity, selectivity and spectrum of hits we obtained. A library of 1725 fragments was tested at a single concentration for growth inhibitory activity against *M. bovis* BCG as screening strain and 38 of 116 primary hits were confirmed in dose response analyses to be active against virulent *M. tuberculosis*. Bacterial kill experiments showed that most hits displayed bactericidal activity at their minimal inhibitory concentration. Cytotoxicity assays established that a large proportion of hits displayed a favorable selectivity index for mammalian cells. Importantly, one third of *M. tuberculosis* active fragments were also active against *M. abscessus* and *M. avium*, two emerging non-tuberculous mycobacterial (NTM) pathogens, opening the opportunity to develop broad spectrum antimycobacterials. Activity determination against Gram positive (*Staphylococcus aureus*) and Gram negative (*Escherichia coli, Klebsiella pneumoniae, Acinetobacter baumannii, Pseudomonas aeruginosa*) bacteria, as well as fungi (*Candida albicans, Cryptococcus neoformans*) showed only a small overlap indicating a generally narrow spectrum of these novel antimicrobial hits for mycobacteria. In conclusion, we carried out the first fragment-based whole cell screen against bacteria and identified a substantial number of hits with excellent physicochemical properties and dual activity against *M. tuberculosis* and NTM pathogens. These hits will now be evaluated in animal models of mycobacterial infection to determine whether any of them can be moved forward as a new antimycobacterial fragment drug candidate.

## Introduction

*Mycobacterium tuberculosis* remains the most deadly bacterial pathogen globally and acquired resistance to current antimycobacterials threatens global tuberculosis control efforts (WHO, 2015). In addition to the tuberculosis pandemic, new mycobacterial diseases are emerging. The number of infections with intrinsically resistant environmental NTM such as *M. abscessus* and *M. avium* are increasing in both developing and developed countries. While prolonged chemotherapies are required for treating *M. avium* infections, lung disease caused by *M. abscessus* is considered incurable (Griffith et al., 2007; Nessar et al., 2012; Aksamit et al., 2014; Park and Olivier, 2015). Hence, there is an urgent medical need for new drugs with new mechanisms of action – and with a *broad* antimycobacterial spectrum of activity.

Current drug discovery strategies are largely based on Paul Ehrlich’s magic bullet ‘one drug–one target’ concept. After a decade of high throughput screening against isolated biochemical targets the antimycobacterial discovery field largely moved back to phenotypic whole cell screens to identify compounds with antimicrobial activity first, then deconvolute the target via selection of spontaneous resistance mutants and whole genome sequencing. This strategic shift is due to the large scale failure of target-based approaches. It turned out that the double membrane-containing mycobacterial cell envelope is a formidable permeability barrier. Enzyme inhibitors identified on isolated targets often do not penetrate this mycobacterial fortress (Payne et al., 2007; Barry et al., 2009; Gwynn et al., 2010). The low permeability of the mycobacterial cell envelope also affects the productivity of phenotypic whole cell screens which use the same standard pharmaceutical chemical libraries employed for target-based screens. Screening those drug-like, rule-of-five compliant (Lipinski, 2000) archives has resulted in very low hit rates (Barry et al., 2009; Campbell, 2011).

Interestingly, critical components of anti-tuberculosis regimens, including the key sterilizing first line drug pyrazinamide, are ‘dirty fragments’: they hit multiple targets, i.e., they are ‘dirty,’ and their molecular weights are in the range of 100–300 g/mol, i.e., they are ‘fragments.’ In addition, these drugs are metabolized inside the tubercle bacillus. Only then, after being ‘activated,’ they exert their antimicrobial activity (Gopal and Dick, 2014). This type of mechanism of action (poly-pharmacology), and physicochemical properties (‘extra’ small and reactive), are at odds with main stream antibacterial drug discovery: attractive leads for medicinal chemistry should inhibit a single target, have a decent size to bind a target with high affinity, and should not be reactive to avoid side effects (Dartois and Barry, 2010).

Fragments came to fame in modern drug discovery due to a novel, ‘fragment-based’ screening approach against molecular targets. This lead finding method makes use of very small, rule-of-3 compliant (Congreve et al., 2003) molecules, with weak binding affinities. Biophysical and structural biology methods are employed to identify such weak binders to a particular target and their modes of binding. The low affinity binders are then extended or combined to develop tight, target-specific, high affinity (nM) compounds. Fragments usually represent attractive starting points for medicinal chemistry as they possess favorable physicochemical and hence pharmacokinetic properties: they are relatively small (<300 g/mol) and rather hydrophilic (clogP < 3) and thus combine attractive water solubility with good absorption and tissue distribution (Scott et al., 2012; Hopkins et al., 2014).

Fragment drugs such as pyrazinamide which are also pro-drugs, are among the most efficacious tuberculosis drugs. Thus hitting multiple targets appears to be an attractive property for antimycobacterials. Furthermore, fragments which are small enough to bind multiple sites are more likely to be accepted as substrate analogs as well and metabolized by the bacterium, again a property that seems to make good antimicrobials for tuberculosis. Being small and moderately lipophilic is critical in achieving *in vivo* exposure and tissue penetration, usually a major hurdle during lead optimization (Dartois and Barry, 2013; Dartois, 2014). In the case of mycobacteria, these physicochemical properties likely also have a positive impact on cellular pharmacokinetics: fragments might more easily penetrate the double membrane mycobacterial cell envelope as porins, the channels spanning the outer membrane, prefer small hydrophilic molecules. Thus, fragments may have multiple advantages over larger molecules: favorable absorption and systemic pharmacokinetic properties, favorable tissue distribution and better bacterial uptake (Gopal and Dick, 2014).

Based on these considerations we decided to screen a fragment library, typically only used for structure guided lead finding, for hits showing whole cell activity against the tubercle bacillus. We identified 38 fragments showing growth inhibitory activity against *M. tuberculosis.* We characterized the hit molecules regarding their bactericidal and cytotoxic activity. Furthermore, the hits were profiled for their antimycobacterial, anti-Gram positive/negative and their antifungal spectrum.

## Materials and Methods

### Assembly of Fragment Library

Fragments were selected based on a computational filtration of 90,000 commercially available molecules from libraries supplied by ChemBridge, Life Chemicals, Enamine, Bionet/Key Organics and Maybridge. The rule-of-3 was applied (Congreve et al., 2003) with additional selections up to molecular weight of 350 for dense functionalities such as sulphonamides. The principle of positive selection of desired hydrogen bonding groups and computational clustering described by Baurin et al. (2004) were also applied to ensure diverse proportional clusters representing a range of polar and solubilising groups. Then a categorization of candidate fragments was made with 0, 1, 2, or 3 H-bond donors, 0, 1, or 2 positively charged nitrogen groups, and up to 1 negatively charged group, the latter being a smaller set. Specific undesired groups were removed such as non-aromatic N–N bonded compounds (e.g., hydrazines), primary anilines, ureas/thioureas, adamantanes, bromo or iodo containing compounds and more than 2 chloro groups. From the approximately 6,000 remaining fragments, further prioritization was carried out with a focus on structures which were not completely flat and had less commonly encountered combinations of functionalities. This resulted in a library of 1725 selected fragment compounds used for whole cell screening described in this report. All fragments are commercially available and the corresponding compound codes of the hits identified in this work are given in Supplementary Table S1.

### Bacterial Strains, Cells, Culture Conditions and Chemicals

*Mycobacterium tuberculosis* H37Rv (ATCC 27294), *M. bovis* BCG (ATCC 35734), *M. abscessus* Bamboo (smooth morphotype) and *M. avium* 11 liquid cultures were grown in Middlebrook 7H9 broth (BD Difco) supplemented with 0.5% albumin, 0.2% glucose, 0.085% sodium chloride, 0.5% glycerol, 0.05% Tween80 as described previously (Lakshminarayana et al., 2015). *Escherichia coli* (ATCC 25922), *Klebsiella pneumoniae* (ATCC 700603, MDR), *Acinetobacter baumannii* (ATCC 19606), *Pseudomonas aeruginosa* (ATCC 27853), *Staphylococcus aureus* (ATCC 43300, MRSA), were cultured in cation-adjusted Mueller Hinton broth at 37°C overnight. *Candida albicans* (ATCC 90028), *Cryptococcus neoformans* (ATCC 208821) were cultured for 3 days on Yeast Extract-Peptone Dextrose agar at 30°C. A yeast suspension of 10^6^ to 5 × 10^6^ cells/mL (as determined by OD530, absorbance at 530 nm) was prepared from five colonies and subsequently used for screening. HepG2 (ATCC HB.8065) and A549 (ATCC CCL-185) cells were cultured at 37°C with 5% CO_2_ atmosphere in DMEM media (Gibco) complemented with 10% FBS heat-inactivated (Gibco), penicillin (100 U/mL, Gibco) and streptomycin (100 μg/mL, Gibco). Red blood cells were obtained from Interstate Blood Bank, Inc. Laboratory, USA. Fragments were obtained from various chemical providers shown in Supplementary Table S1. All experiments on *M. tuberculosis* were conducted in a BSL-3 core facility following biosafety level 3 procedures.

### Antimicrobial and Antifungal Assays

Antimycobacterial screens were carried out as follows. All mycobacterial precultures were harvested at mid-log phase and diluted to an OD600 (absorbance at 600 nm) of 0.05 in complete 7H9 medium. *M. bovis* BCG bacterial suspensions were then dispensed into 96-well plates (200 μL/well) with a single concentration of 500 μM of fragment per well in the primary screen. Plates were incubated for 5 days at 37°C under shaking (100 rpm). Compounds causing growth inhibition >80% as compared to untreated control in the same plate were considered positive hits. Cells were manually resuspended and OD was measured at 600 nm on M200Pro plate reader (Tecan) (Murugasu-Oei and Dick, 2000; Gopal and Dick, 2015; Lakshminarayana et al., 2015). Hits that were re-confirmed in the same assay from re-ordered fresh powder stock were then tested against *M. tuberculosis* in a dose-response format (twofold serial dilution from a maximum concentration of 500 μM). Active hits were subsequently tested against *M. abscessus* and *M. avium* in a similar manner. *M. abscessus* and *M. avium* containing 96-well plates were incubated for 3 and 5 days respectively at 37°C under shaking (100 rpm). Ten micromolar ciprofloxacin was used as a positive control for *M. tuberculosis* and *M. bovis* BCG. Hundred micromolor kanamycin was used as a positive control for *M. abscessus* and *M. avium*. Bactericidal activity against *M. bovis* BCG was determined by exposing approximately 10^6^ CFU to MIC_90_ concentration for 5 days. CFU were then recovered on agar plates for enumeration and compared to CFU recovered from inoculum as described previously (Murugasu-Oei and Dick, 2000; Lakshminarayana et al., 2015). Fold-kill was calculated as the reduction in CFU in treated samples as compared to inoculum. All experiments were carried out two times independently and mean values are shown. Standard deviations were smaller than 20%.

Antimicrobial screening against gram positive and negative bacteria as well as fungi was performed by the Community for Antimicrobial Drug Discovery, University of Queensland, funded by the Wellcome Trust (UK) and The University of Queensland (Australia). A sample of an overnight culture of the respective test bacterium was diluted 40-fold in fresh broth and incubated at 37°C for 1.5–3 h. The resultant mid-log phase cultures were diluted (CFU/mL measured by OD600), then 45 μL was added to each well of the compound containing plates, giving a cell density of 5 × 10^5^ CFU/mL. The ten best compounds were tested in a twofold serial dilution format at a maximum concentration of 100 μM. All the plates were covered and incubated at 37°C for 18 h without shaking. Inhibition of bacterial growth was determined measuring absorbance at 600 nm (OD600), using a Tecan M1000 Pro monochromator plate reader. The percentage of growth inhibition was calculated for each well, using the negative control (media only) and positive control (bacteria without inhibitors) on the same plate as references. Colistin and Vancomycin were used as positive bacterial inhibitor standards for Gram negative and Gram-positive bacteria, respectively, at four concentrations including two above and two below the MIC of the respective pathogens. All experiments were carried out two times independently and mean values are shown. Standard deviations were smaller than 20%.

Fungal stock suspensions described above were diluted with Yeast Nitrogen Base broth to a final concentration of 2.5 × 10^3^ CFU/mL. Then, 45 μL of the fungi suspension was added to each well of the compound-containing plates. The ten best compounds were tested in a twofold serial dilution format at a maximum concentration of 100 μM. Plates were covered and incubated at 35°C for 24 h without shaking. Growth inhibition of *C. albicans* was determined measuring absorbance at 530 nm (OD530), while the growth inhibition of *C. neoformans* was determined measuring the difference in absorbance between 600 and 570 nm (OD600-570), after the addition of resazurin (0.001% final concentration) and incubation at 35°C for additional 2 h. The absorbance was measured using a Biotek Synergy HTX plate reader. The percentage of growth inhibition was calculated for each well, using the negative control (media only) and positive control (fungi without inhibitors) on the same plate. Fluconazole was used as a positive fungal inhibitor standard for *C. albicans* and *C. neoformans* at four concentrations including two above and two below the MIC. All experiments were carried out two times independently and mean values are shown. Standard deviations were smaller than 20%.

### Cytotoxicity Assays

The MTS [3-(4,5-dimethylthiazol-2-yl)-2,5-diphenyltetrazolium bromide] reduction assay (Promega) was used according to manufacturer’s guidelines to assess HepG2 or A549 cell viability after exposure to a range of compound concentrations (twofold serial dilution) (Riss et al., 2004). Absorbance was read with a Tecan M200Pro plate reader at 570 nm. Hundred micromolor of carbonyl cyanide *m*-chlorophenylhydrazone was used as a cytotoxic positive control. Hemolytic activity was evaluated by exposing 10^6^ red blood cells to two-fold serial dilutions of compounds in phosphate-buffered saline for 24 h. Red blood cells were subsequently centrifuged at 1000 *g* for 10 min and the supernatant was collected and homogenized by vortexing. Absorbance was read at 540 nm. A Triton X-100 treated control (2% Triton) was used to define 100% of hemoglobin release (Dausset and Contu, 1967). All experiments were carried out two times independently and mean values are shown. Standard deviations were smaller than 20%.

### Physico-Chemical Characterization of Hits

Molecular properties of the fragment hits were calculated using Molinspiration software^1^. Hydrogen bond donors are defined as the total number of OH + NH groups. Hydrogen bond acceptors are defined as the O + N count. Calculated LogP (cLogP) was determined using the miLogP2.2 (November 2005) software from Molinspiration. Topological Polar Surface Area (TPSA) (Ertl et al., 2000) is defined as the sum of the surfaces of the polar atoms in a molecule. Number of rotatable bonds (NROT) is defined as the count of any single non-ring bond, bounded to a non-hydrogen atom; NROT does not include amide bonds which are considered non-rotatable.

## Results

### Identification of Hits Active against *M. tuberculosis* from a Fragment Library

A library of 1725 fragments was carefully selected out of a collection of 90,000 commercially available fragments based on their physicochemical and chemical properties (see Material and Methods). We first screened the library against our biosafety level 2 screening strain *M. bovis* BCG as a surrogate for *M. tuberculosis* in order to identify hits with whole-cell activity.

**FIGURE 1:**
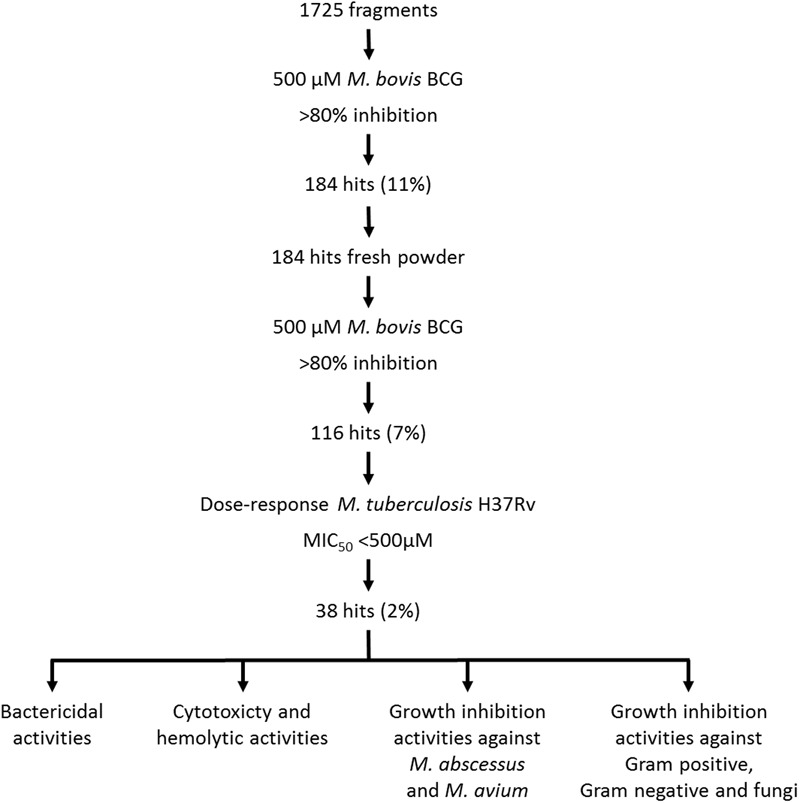
**Screening cascade and hit characterization. (Upper part)** Screening cascade with associated number of hits. **(Bottom part)** Various hits characterization activities. For details see Results.

**FIGURE 2:**
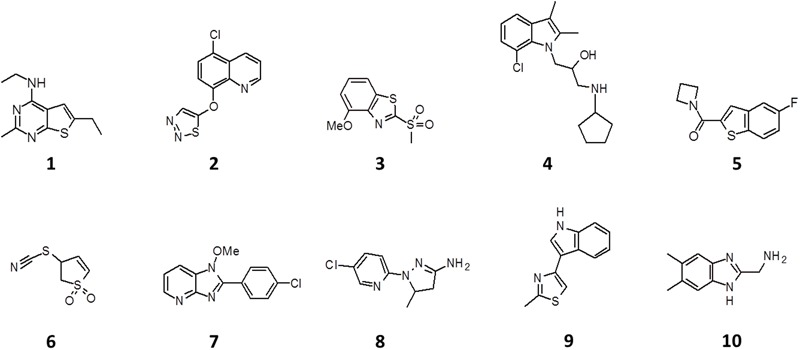
**Structure of the 10 most potent *M. tuberculosis* actives.** See **Table 1** for antibacterial, antifungal and cytotoxic activities.

The screen was carried out using a single concentration of 500 μM of the test compounds. Growth inhibition of at least 80% was defined as cut-off which resulted in 184 primary hits (11% hit rate). These hits were re-purchased as powders and the compounds were re-tested in the same single point 500 μM assay resulting in 116 confirmed hits (7% hit rate). For these 116 hits, growth inhibition dose response was measured against *M. tuberculosis* and compounds with an MIC_50_ < 500 μM were defined as whole cell actives, resulting in 38 hits (2% hit rate). A cut-off of 500 μM was derived from the MIC_50_ of the anti-tuberculosis fragment drug pyrazinamide which was selected as a benchmark (Via et al., 2015). The screening cascade and hit confirmation is depicted in **Figure 1**. **Figure 2** and **Table 1** show the structures and the MIC_50_ of the most potent *M. tuberculosis* active hits. **Table 2** describes in detail their physicochemical properties. The structures and activities of all 38 hits are shown in Supplementary Table S1.

**Table 1:** Antibacterial, antifungal and cytotoxic activities of the 10 most potent *M. tuberculosis* actives.

	*M. tuberculosis*	*M. bovis* BCG		*M. abscessus*	*M. avium*	*S. aureus*	*E. coli*	*K. pneumoniae*	*A. baumannii*	*P. aeruginosa*	*C. albicans*	*C. neoformans*	HepG2		A549		RBC	
#	MIC_50_	MIC_50_	Fold kill at MIC_90_^a^	MIC_50_		Growth Inhibition Spectrum (MIC_80_)^b^							CC_50_^c^	SI	CC_50_^c^	SI	HC_50_^c^	SI
1	40	5	100	>500	>500	>100	>100	>100	>100	>100	>100	>100	i	>24	i	>24	i	>24
2	43	56	100	>500	130	>100	>100	>100	>100	>100	>100	>100	200	5	i	>7	250	6
3	52	20	nd	>500	>500	>100	>100	>100	>100	>100	>100	>100	nd	nd	nd	nd	i	>13
4	55	56	100	125	53	>100	>100	>100	>100	>100	>100	>100	45	1	60	1	125	2
5	60	68	10	>500	>500	>100	>100	>100	>100	>100	>100	>100	i	>41	i	>41	i	>41
6	92	53	100	>500	>500	100	>100	>100	100	>100	100	>100	75	1	1000	11	2200	24
7	104	19	10	150	145	>100	>100	>100	>100	>100	>100	>100	850	8	1800	17	i	>23
8	123	13	100	>500	275	>100	100	>100	100	>100	100	>100	900	7	i	>20	i	>20
9	126	39	10	>500	>500	>100	>100	>100	>100	>100	>100	>100	500	4	i	>20	i	>18
10	131	53	10	>500	>500	>100	>100	>100	>100	>100	>100	>100	900	7	1900	15	2100	16

*Activities of hits against mycobacteria, Gram positive and negative bacteria, fungi and mammalian cells are shown. Concentrations are reported in micromolar (μM). MIC_*50*_, MIC_*80*_, MIC_*90*_, minimum inhibitory concentration that inhibits 50, 80, and 90% of bacterial growth, respectively. MIC_*90*_ were in general 1–5 × MIC_*50*_. CC_*50*_, HC_*50*_, cytotoxic and hemolytic concentrations that kill or lyse 50% of cells as compared to untreated control, respectively. SI, selectivity index as defined by CC_*50*_ or HC_*50*_/MIC_*50*_. RBC, red blood cells. nd, not determined. ^a^fold-kill as compared to untreated control; ^b^all top 10 hits showed an MIC_*80*_ ≥ 32 μg/mL (or approximately 100 μM), the highest concentration tested; ^c^i, inactive denotes no cytotoxic or hemolytic activity at five times the MIC_90_; All experiments were carried out two times independently and mean values are shown. Standard deviations were smaller than 20%.*

**Table 2:** Physico-chemical properties of the 10 most potent *M. tuberculosis* actives.

Fragments #	MW	HBD^1^	HBA^2^	clogP^3^	TPSA^4^	N_ROT_^5^
1	221.3	1	3	2.54	37.8	3
2	263.7	0	4	3.30	47.9	2
3	243.3	0	4	1.98	56.3	2
4	320.9	2	3	4	37.2	5
5	235.3	0	2	1.88	20.3	1
6	175.2	0	3	0.04	57.9	1
7	259.7	0	4	3.35	40.0	2
8	210.7	2	4	1.76	54.5	1
9	214.3	1	2	2.41	28.7	1
10	175.2	3	3	1.52	54.7	1

*MW, molecular weight; HBD, Hydrogen Bond Donor; HBA, Hydrogen Bond Acceptor; ^1^Properties calculated using Molinspiration software (www.molinspiration.com); HBD, OH + NH count. ^2^HBA, O + N count. cLogP, partition coefficient between *n*-octanol and water log; ^3^cLogP, calculated LogP (miLogP2.2 – November 2005). ^4^TPSA, Topological Polar Surface Area. ^5^N_ROT_, number of rotatable bonds.*

### Bactericidal Activities and Cytotoxicity of Hits

To determine the bactericidal activities of the hits we exposed tubercle bacilli to MIC_90_ concentrations of the respective compounds and counted CFU after 5 days of treatment. **Table 1**; Supplementary Table S1 shows that more than half of the hits reduced the number of CFU at least 100-fold showing that most fragment hits displayed strong cidal activity. To evaluate *in vitro* tolerability of the 38 *M. tuberculosis* actives we measured their cytotoxicity against two mammalian cell lines as well as their membrane toxicity using a red blood cell lysis assay. **Table 1**; Supplementary Table S1 show that half of the hits showed acceptable cytotoxic and hemolytic activity with a selectivity index of more than 5.

### Activity of *M. tuberculosis* Actives against *M. abscessus* and *M. avium*

**FIGURE 3:**
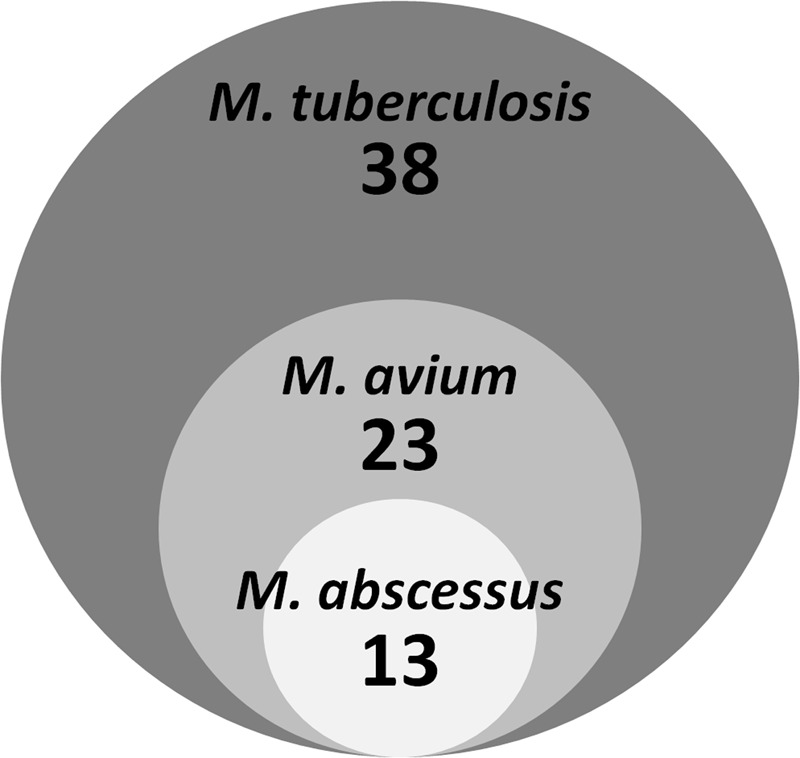
**Activity of *M. tuberculosis* actives against *M. avium* and *M. abscessus*.** The diagram shows overlapping activity of 38 *M. tuberculosis* hits against two non-tuberculous mycobacterial species.

To establish the spectrum of activity of the 38 *M. tuberculosis* active hits against other mycobacteria we measured their growth inhibition activity against the fast growing *M. abscessus* and the slow growing *M. avium.* **Table 1**; Supplementary Table S1 shows that two thirds of the *M. tuberculosis* actives were also active against *M. avium*, while one third showed activity against *M. abscessus*. **Figure 3** shows that the 13 *M. abscessus* active hits in fact represent a subset of the *M. avium* actives.

### Activity of *M. tuberculosis* Actives against Gram Positive and Gram Negative Pathogens and Fungi

To examine whether the *M. tuberculosis* actives have a mostly *Mycobacterium*-specific range of activity or tend to display broad spectrum activity we determined their growth inhibition against Gram positive (*Staphylococcus aureus*) and negative (*Escherichia coli, Klebsiella pneumoniae, Acinetobacter baumannii, Pseudomonas aeruginosa*) bacteria, as well as fungi (*Candida albicans, Cryptococcus neoformans*). **Table 1**; Supplementary Table S1 show that only a small overlap was observed indicating a generally narrow spectrum of the fragment hits for mycobacteria.

## Discussion

Based on the success of pyrazinamide and other ‘dirty’ fragment drugs in the treatment of tuberculosis patients, we previously proposed that fragment-based whole cell screens, currently not part of mainstream drug discovery efforts, should be included in antimycobacterial discovery activities (Gopal and Dick, 2014). Here we carried out such a screen of 1725 compounds and identified 38 rule-of-3 compliant fragments (hit rate 2%) with growth inhibitory activity against *M. tuberculosis*. When compared to the hit rate of standard pharmaceutical libraries (<0.5%; Campbell, 2011) this higher hit rate is consistent with our expectation that small hydrophilic fragment compounds should have favorable bacterial cell penetration properties compared to larger and more hydrophobic molecules (Gopal and Dick, 2014). Interestingly, most hits showed strong mycobactericidal activity while a large fraction retained an acceptable selectivity index for mammalian cells. All hits displayed excellent physic-chemical properties and low molecular weight but no particular structure-activity relationship could be established. General pathogen spectrum profiling of the 38 anti-tuberculosis hits against Gram positive and negative bacteria and against fungi revealed a limited overlap suggesting that most of the hits are rather *Mycobacterium*-specific.

Importantly, a substantial proportion of *M. tuberculosis* hits also exhibited activity against NTM pathogens, specifically *M. abscessus* and *M. avium*. New drugs to treat lung disease caused by NTM represent an new urgent medical need as *M. avium* causes a large number of infections requiring prolonged treatments, and *M. abscessus* infections, although smaller in number, are considered incurable (Griffith, 2011). The identification of hits active against both *M. tuberculosis* and NTM opens the opportunity to progress anti *M. tuberculosis* hits as broad spectrum antimycobacterials.

The identified antimycobacterial fragment hits will now be moved forward following two different strategies. Firstly, we will ask whether we can ‘identify the next pyrazinamide,’ i.e., a new antimycobacterial fragment drug. To explore this possibility we will directly move hits into efficacy studies using mouse models of mycobacterial infection, after selecting a subset of compounds showing acceptable *in vivo* tolerability and pharmacokinetics. If metabolic stability turns out to be an issue, minor chemical modifications will be carried out to determine whether this property can be improved without affecting potency. The objective of this first strategy is to determine whether any of the fragment hits can be moved more or less directly, i.e., without a major lead optimization program, to preclinical development as a fragment drug (Dick et al., 2007). In a second strategy we ask whether we can use any of these rule-of-3 compliant fragment hits to generate a more standard drug-like molecule, i.e., a rule-of-5 compliant, high affinity, high potency, single target compound. Due to their fragment nature, all hits have excellent physico-chemical properties and hence represent attractive starting points for lead optimization campaigns.

In parallel to the two pronged drug discovery program, the mechanism of action of the fragment hits will be determined. It appears likely, due to their nature as promiscuous binders, that the new antimycobacterials have multiple targets, i.e., that they act as ‘dirty’ drugs. Furthermore, due to their potential to act as enzyme substrates, and similarly to other anti-TB fragment drugs such as isoniazid (INH), ethionamide (ETH), para-amino salicylic acid (PAS) or pyrazinamide (PZA), it is likely that a subset of the 38 fragment hits will be metabolized and ‘activated’ by the bacilli, i.e., that they act as reactive ‘dirty’ drugs (Gopal and Dick, 2014). Dissecting the mechanisms of action of existing tuberculosis fragment drugs resulted in the identification of several novel targets and novel concepts of antimicrobial action (Gopal and Dick, 2014). Hence we expect that the mechanism of action studies of this collection of novel, mostly bactericidal fragments may uncover novel antimicrobial concepts.

## Conclusion

We carried out the first fragment-based whole cell screen against mycobacteria and identified a substantial number of hits with excellent physicochemical properties which represent a rich source for antimycobacterial drug discovery and chemical biology projects.

## Author Contributions

WM and TD conceived and designed the project. WM, JJL, SYY, and PMR carried out the experiments. WM, BWD, and TD wrote the manuscript.

## Conflict of Interest Statement

The authors declare that the research was conducted in the absence of any commercial or financial relationships that could be construed as a potential conflict of interest.

## Footnotes

1 http://www.molinspiration.com

## Abbreviations

CFU: colony forming units
NTM: non-tuberculous mycobacteria

## References

[B1] Aksamit T. R. Philley J. V. Griffith D. E. (2014). Nontuberculous mycobacterial (NTM) lung disease: the top ten essentials. *Respir. Med.* 108 417–425. 10.1016/j.rmed.2013.09.014 24484653

[B2] Barry C. E. III Boshoff H. I. Dartois V. Dick T. Ehrt S. Flynn J. (2009). The spectrum of latent tuberculosis: rethinking the biology and intervention strategies. *Nat. Rev. Microbiol.* 7 845–855. 10.1038/nrmicro2236 19855401 PMC4144869

[B3] Baurin N. Aboul-Ela F. Barril X. Davis B. Drysdale M. Dymock B. (2004). Design and characterization of libraries of molecular fragments for use in NMR screening against protein targets. *J. Chem. Inf. Comput. Sci.* 44 2157–2166. 10.1021/ci049806z 15554686

[B4] Campbell J. (2011). High-throughput assessment of bacterial growth inhibition by optical density measurements. *Curr. Protoc. Chem. Biol.* 3:100115 10.1002/9780470559277.ch100115 PMC3182142 21966637

[B5] Congreve M. Carr R. Murray C. Jhoti H. (2003). A ‘rule of three’ for fragment-based lead discovery? *Drug Discov. Today* 8 876–877. 10.1016/S1359-6446(03)02831-9 14554012

[B6] Dartois V. (2014). The path of anti-tuberculosis drugs: from blood to lesions to mycobacterial cells. *Nat. Rev. Microbiol.* 12 159–167. 10.1038/nrmicro3200 24487820 PMC4341982

[B7] Dartois V. Barry C. E. (2010). Clinical pharmacology and lesion penetrating properties of second- and third-line antituberculous agents used in the management of multidrug-resistant (MDR) and extensively-drug resistant (XDR) tuberculosis. *Curr. Clin. Pharmacol.* 5 96–114. 10.2174/157488410791110797 20156156 PMC6344931

[B8] Dartois V. Barry C. E. III. (2013). A medicinal chemists’ guide to the unique difficulties of lead optimization for tuberculosis. *Bioorg. Med. Chem. Lett.* 23 4741–4750. 10.1016/j.bmcl.2013.07.006 23910985 PMC3789655

[B9] Dausset J. Contu L. (1967). Drug-induced hemolysis. *Annu. Rev. Med.* 18 55–70. 10.1146/annurev.me.18.020167.000415 5337612

[B10] Dick T. Dartois V. Keller T. Matter A. (2007). “TB drug discovery from target identification to proof of concept studies,” in *Handbook of Tuberculosis: Clinics, Diagnostics, Therapy and Epidemiology* eds Kaufmann S. H. E. van Helden P. (Weinheim: Wiley-VCH) 139–159.

[B11] Ertl P. Rohde B. Selzer P. (2000). Fast calculation of molecular polar surface area as a sum of fragment-based contributions and its application to the prediction of drug transport properties. *J. Med. Chem.* 43 3714–3717. 10.1021/jm000942e 11020286

[B12] Gopal P. Dick T. (2014). Reactive dirty fragments: implications for tuberculosis drug discovery. *Curr. Opin. Microbiol.* 21 7–12. 10.1016/j.mib.2014.06.015 25078318

[B13] Gopal P. Dick T. (2015). The new tuberculosis drug Perchlozone^®^ shows cross-resistance with thiacetazone. *Int. J. Antimicrob. Agents* 45 430–433. 10.1016/j.ijantimicag.2014.12.026 25704063

[B14] Griffith D. E. (2011). The talking *Mycobacterium abscessus* blues. *Clin. Infect. Dis.* 52 572–574. 10.1093/cid/ciq252 21292660

[B15] Griffith D. E. Aksamit T. Brown-Elliott B. A. Catanzaro A. Daley C. Gordin F. (2007). An official ATS/IDSA statement: diagnosis, treatment, and prevention of nontuberculous mycobacterial diseases. *Am. J. Respir. Crit. Care Med.* 175 367–416. 10.1164/rccm.200604-571ST 17277290

[B16] Gwynn M. N. Portnoy A. Rittenhouse S. F. Payne D. J. (2010). Challenges of antibacterial discovery revisited. *Ann. N. Y. Acad. Sci.* 1213 5–19. 10.1111/j.1749-6632.2010.05828.x 21058956

[B17] Hopkins A. L. Keseru G. M. Leeson P. D. Rees D. C. Reynolds C. H. (2014). The role of ligand efficiency metrics in drug discovery. *Nat. Rev. Drug Discov.* 13 105–121. 10.1038/nrd4163 24481311

[B18] Lakshminarayana S. B. Huat T. B. Ho P. C. Manjunatha U. H. Dartois V. Dick T. (2015). Comprehensive physicochemical, pharmacokinetic and activity profiling of anti-TB agents. *J. Antimicrob. Chemother.* 70 857–867. 10.1093/jac/dku457 25587994 PMC7714050

[B19] Lipinski C. A. (2000). Drug-like properties and the causes of poor solubility and poor permeability. *J. Pharmacol. Toxicol. Methods* 44 235–249. 10.1016/S1056-8719(00)00107-6 11274893

[B20] Murugasu-Oei B. Dick T. (2000). Bactericidal activity of nitrofurans against growing and dormant *Mycobacterium bovis* BCG. *J. Antimicrob. Chemother.* 46 917–919. 10.1093/jac/46.6.917 11102410

[B21] Nessar R. Cambau E. Reyrat J. M. Murray A. Gicquel B. (2012). *Mycobacterium abscessus*: a new antibiotic nightmare. *J. Antimicrob. Chemother.* 67 810–818. 10.1093/jac/dkr578 22290346

[B22] Park I. K. Olivier K. N. (2015). Nontuberculous mycobacteria in cystic fibrosis and non-cystic fibrosis bronchiectasis. *Semin. Respir. Crit. Care Med.* 36 217–224. 10.1055/s-0035-1546751 25826589 PMC7171444

[B23] Payne D. J. Gwynn M. N. Holmes D. J. Pompliano D. L. (2007). Drugs for bad bugs: confronting the challenges of antibacterial discovery. *Nat. Rev. Drug Discov.* 6 29–40. 10.1038/nrd2201 17159923

[B24] Riss T. L. Moravec R. A. Niles A. L. Benink H. A. Worzella T. J. Minor L. (2004). “Cell viability assays,” in *Assay Guidance Manual* eds Sittampalam G. S. Coussens N. P. Nelson H. Arkin M. Auld D. Austin C. (Rockville, MD: Bethesda).

[B25] Scott D. E. Coyne A. G. Hudson S. A. Abell C. (2012). Fragment-based approaches in drug discovery and chemical biology. *Biochemistry* 51 4990–5003. 10.1021/bi3005126 22697260

[B26] Via L. E. Savic R. Weiner D. M. Zimmerman M. D. Prideaux B. Irwin S. M. (2015). Host-mediated bioactivation of pyrazinamide: implications for efficacy, resistance, and therapeutic alternatives. *ACS Infect. Dis.* 1 203–214. 10.1021/id500028m 26086040 PMC4467917

[B27] WHO (2015). *Global Tuberculosis Report 2015.* Geneva: World Health Organization.

